# Accuracy of Estimation of Graft Size for Living-Related Liver Transplantation: First Results of a Semi-Automated Interactive Software for CT-Volumetry

**DOI:** 10.1371/journal.pone.0110201

**Published:** 2014-10-17

**Authors:** Theresa Mokry, Nadine Bellemann, Dirk Müller, Justo Lorenzo Bermejo, Miriam Klauß, Ulrike Stampfl, Boris Radeleff, Peter Schemmer, Hans-Ulrich Kauczor, Christof-Matthias Sommer

**Affiliations:** 1 Department of Diagnostic and Interventional Radiology, University Hospital Heidelberg, Heidelberg, Germany; 2 Philips Healthcare Germany, Hamburg, Germany; 3 Department of Medical Biometry and Informatics, University Hospital Heidelberg, Heidelberg, Germany; 4 Department of General and Transplant Surgery, University Hospital Heidelberg, Heidelberg, Germany; University of Pittsburgh, United States of America

## Abstract

**Objectives:**

To evaluate accuracy of estimated graft size for living-related liver transplantation using a semi-automated interactive software for CT-volumetry.

**Materials and Methods:**

Sixteen donors for living-related liver transplantation (11 male; mean age: 38.2±9.6 years) underwent contrast-enhanced CT prior to graft removal. CT-volumetry was performed using a semi-automated interactive software (P), and compared with a manual commercial software (TR). For P, liver volumes were provided either with or without vessels. For TR, liver volumes were provided always with vessels. Intraoperative weight served as reference standard. Major study goals included analyses of volumes using absolute numbers, linear regression analyses and inter-observer agreements. Minor study goals included the description of the software workflow: degree of manual correction, speed for completion, and overall intuitiveness using five-point Likert scales: 1–markedly lower/faster/higher for P compared with TR, 2–slightly lower/faster/higher for P compared with TR, 3–identical for P and TR, 4–slightly lower/faster/higher for TR compared with P, and 5–markedly lower/faster/higher for TR compared with P.

**Results:**

Liver segments II/III, II–IV and V–VIII served in 6, 3, and 7 donors as transplanted liver segments. Volumes were 642.9±368.8 ml for TR with vessels, 623.8±349.1 ml for P with vessels, and 605.2±345.8 ml for P without vessels (P<0.01). Regression equations between intraoperative weights and volumes were y = 0.94x+30.1 (R^2^ = 0.92; P<0.001) for TR with vessels, y = 1.00x+12.0 (R^2^ = 0.92; P<0.001) for P with vessels, and y = 1.01x+28.0 (R^2^ = 0.92; P<0.001) for P without vessels. Inter-observer agreement showed a bias of 1.8 ml for TR with vessels, 5.4 ml for P with vessels, and 4.6 ml for P without vessels. For the degree of manual correction, speed for completion and overall intuitiveness, scale values were 2.6±0.8, 2.4±0.5 and 2.

**Conclusions:**

CT-volumetry performed with P can predict accurately graft size for living-related liver transplantation while improving workflow compared with TR.

## Introduction

Computer-assisted image analysis is an emerging technology for diagnosis, therapy and follow-up in making observer-independent and reproducible readings, and can improve the workflow compared with conventional image analysis. Time-efficient image post-processing with correct interpretation is of great importance, considering the increasing speed of data acquisition on the one hand and the extremely large amount of data available for interpretation on the other. Computer-assisted image analysis is a special challenge for the liver because of motion and deformation during respiration, multi-phase image acquisition and segmental anatomy with four different tubular systems. Although multiple approaches for computer-assisted image analysis have been introduced for oncologic liver resection, living-related liver transplantation and interventional oncology there seems to be still a lack of satisfactory solutions for the clinical routine [1]. For patients with end-stage liver disease, liver transplantation is the most effective treatment [2]. The great increase in the number of patients awaiting liver transplantation during the past years has led to a significant shortage of cadaveric organs [3]. Living-related liver transplantation has emerged as a valuable alternative, and allows healthy adults to donate a part of their own liver to a compatible recipient [4], [5]. Since the convincing results associated with low risk for the healthy donor and improved outcome for the diseased recipient compared with cadaveric liver transplantation, living-related liver transplantation becomes more and more common [6], [7]. For the clinical success after living-related liver transplantation, the liver volume plays a key role [8], [9]. For donor and recipient, the post-operative liver volume must be large enough to fulfill metabolic demands [10]. Additionally, the liver graft should not be oversized since compression can lead to liver necrosis and impaired wound healing with potentially fatal outcome for the recipient (large-for-size) [11]. Currently, non-automated CT-volumetry can be regarded as the preoperative standard to assess liver anatomy and the future graft size [12]. After clinical estimation of the adequate graft size for the recipient (e.g. using the “graft weight to body weight ratio”), the most suitable segments for liver donation can be defined on the basis of CT-volumetry [13]–[15]. With this background, we defined the objective of our study: to evaluate accuracy of estimation of graft size for living-related liver transplantation using a semi-automated interactive software for CT liver volumetry (P). We hypothesized that CT-volumetry performed with P can predict accurately graft size while improving workflow compared with a manual commercial software (TR).

## Materials and Methods

### Ethics Statement

The Institutional Review Board (Ethikkommission der Medizinischen Fakultät Heidelberg, Heidelberg, Germany) approved the study. Every donor underwent an individual standardized evaluation process for living-related liver donation in regards of ethical as well as medical issues. Written informed consent was obtained for all donors. The data was analyzed retrospectively from a prospective digital database.

### Donors for Living-Related Liver Transplantation

From January 2008 until December 2011, donors for living-related liver transplantation were enrolled. Inclusion criteria were (I) typical liver resection for living-related liver donation as well as (II) CT examination according to our standard protocol “living-related liver donor evaluation”. Sixteen donors (11 male) with a mean age of 38.2±9.6 years were identified. All donors were healthy adults. Suitability for living-related liver donation was approved according to standard operating procedures including history, clinical examination, blood analysis, echocardiography, lung function, chest x-ray, and psychosocial evaluation [16]. Detailed patient demographics are presented in Table 1.

**Table 1:** Patient Demographics.

Parameter	Study group
Age (years)	38.2±9.6 (19–63)*
Gender (male/female)	11/5
Height (cm)	173.4±10.5 (148–189)*
Weight (kg)	74.3±13.4 (53–105)*
BMI (kg/m^2^)	24.6±2.9 (20–32)*
BSA (m^2^)	1.9±0.2 (1.48–2.29)*

Note: given numbers are mean±SD (range).

### CT Examination

A 64-row multi-detector CT scanner (Somatom Definition DS, Siemens Medical Solutions, Forchheim, Germany) was used. The CT scan protocol consisted of multiple different phases of the liver: non-enhanced, biliary, arterial, and portal-venous. A late phase was acquired in the case of focal lesions. Prior to acquisition of the non-enhanced phase, all donors received an intravenous premedication consisting of 4 mg clemastine fumarate (Tavegil; Novartis, Basel, Switzerland) and 20 mg ranitidine hydrochloride (Ranitic; Hexal, Holzkirchen, Germany) to prevent potential adverse effects to the intravenous contrast materials. After continuous intravenous infusion of 100 ml biliary contrast material at a flow rate of 150 ml/h (iotroxate dimeglumine, Biliscopin; Bayer Schering Pharma, Berlin, Germany), the biliary phase was obtained. After intravenous injection of 100 ml of iodinated contrast material at a flow rate of 5 ml/s (iomeprol, Imeron 350; Bracco, Konstanz, Germany), arterial and portal-venous phases were acquired. Automated bolus tracking in the aorta at the level of the celiac trunc ensured accurate timing of the arterial phase (trigger threshold of 100 HU). The portal-venous phase was obtained with a delay of 50 s. The optional late phase was acquired with an additional delay of 180 s. Major scanning parameters included a tube voltage of 120 kVp, a reference current-time product of 240 mAs (CARE Dose 4D), a pitch of 0.55 and a collimation of 64×0.6 mm. Raw-data of all phases were reconstructed to obtain transverse and coronal images with a slice thickness of 3 mm and an increment of 1 mm, as well as transverse and coronal images with a slice thickness of 1 mm and an overlap of 0.7 mm. All reconstructions were performed in a medium soft tissue kernel (B30f, Siemens Medical Solutions, Siemens, Forchheim). All phases were used to study the liver, and no relevant anatomical variants or pathological conditions (e.g. focal liver lesions) were detected.

### CT-Volumetry

**Figure 1:**
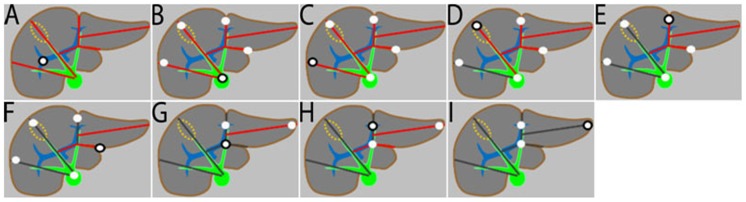
Semi-automated Interactive Software for CT-volumetry (P) – Manual Positioning of 9 Anatomical Landmarks to Define the Segments of Couinaud (Schematic Illustration; Courtesy of Philips Healthcare Germany, Hamburg, Germany). A first bifurcation of the right portal vein (black circle). B inferior caval vein (black circle). C right hepatic vein (black circle). D middle hepatic vein (black circle). E left hepatic vein (black circle). F superficial ligamentum venosum (black circle). G deep ligamentum venosum (black circle). H end of left portal vein (black circle). I left liver tip (black circle) Note: after automated outline of the entire liver with correction of false-positive and false-negative extractions, and then after manual positioning of the 9 anatomical landmarks, volumes of transplanted liver segments are obtained.

Transverse images of the portal-venous phase (slice thickness of 3 mm and increment of 1 mm) were used for CT-volumetry. Two different software tools were used and compared: a semi-automated interactive commercial software called “IntelliSpace Portal Liver Analysis application” (Philips Medical Systems, Best, The Netherland) (P) and a manual commercial software (TR; Aquarius iNtuition; TeraRecon, Foster City, USA). For P, the images were uploaded, and then the outline of the entire liver was determined between liver tissue and surrounding fatty tissue. The algorithm responsible for the segmentation of the liver in contrast-emhanced CT images belongs to the family of variational approaches. It is based on a deformable mesh guided by Hounsfield units as well as surrounding anatomical structures. The algorithm is composed of four different steps. (1) Surrounding anatomical structures are coarsely segmented to provide spatial context. (2) A region inside the liver is localized. (3) Liver tissue likelihood is estimated and refined as the mesh evolves. (4) The mesh is evolved based on likelihood and proximity to surrounding structures. False-positive and false-negative extractions could be corrected using manual correction tools. After manual positioning of 9 anatomical landmarks proposed by the software using the “work-me-through” tool in the “landmark selection mode”, the segments of Couinaud were then calculated automatically, and volumes of transplanted liver segments were obtained subsequently (Fig. 1). Since the opportunity to segment automatically liver veins and portal veins between liver tissue and vasculature, P provided liver volumes with and without vessels. For TR, the images were uploaded in the ‘CTA Abdomen’ workflow. Using the free region-of-interest (ROI) tool, the outline of the entire liver and transplanted liver segments were set manually on every image slice, and respective liver volumes were provided always with vessels. A forth study group with TR without vessels was not performed since the proceeding would have been extremely time consuming.

### Weight of Transplanted Liver Segments

The intraoperative weight of transplanted liver segments was defined as reference standard for the graft size. After resection of the respective liver segments, the grafts were flushed with normal saline to remove the blood. Grafts were prepared and weighed on the back table with a precision balance with an accuracy of 0.5 g.

### Study Goals, Data Acquisition, and Statistical Analysis

The primary study goal was the definition of volumes of transplanted liver segments. Two observers (Observer 1 (T.M.) and Observer 2 (N.B.) with 1 and 3 years experience with preoperative CT-volumetry, respectively) independently performed CT-volumetry twice (interval between both reads>30 days). Consequently, 4 reads (Read 1 and Read 2 for Observer 1 as well as Read 1 and Read 2 for Observer 2) were available for P with vessels, P without vessels, and TR with vessels. To describe statistically significant differences of volume of transplanted liver segments between the 3 different techniques, ANOVA for repeated measures was applied. Linear regression analysis between intraoperative weights and volumes was performed with volume on the x-axis and intraoperative weight on the y-axis. Disagreement between intraoperative weights and volumes was calculated as published previously [3]: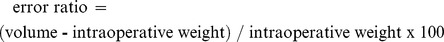.

To describe statistically significant differences for the error ratio between the 3 different techniques, ANOVA for repeated measures was applied. Intra-observer and inter-observer agreements of volumes were calculated applying the Blant-Altman analysis with bias and 95% limits of agreement. The secondary study goal was to describe the software workflow. The degree of manual correction during CT-volumetry was rated for each read applying a five-point Likert scale: 1–markedly lower for P compared with TR, 2–slightly lower for P compared with TR, 3–identical for P and TR, 4–slightly lower for TR compared with P, and 5–markedly lower for TR compared with P. The speed for completion of CT-volumetry (from the start of the uploading of the images to the final report) was rated for each read applying a five-point Likert scale: 1–markedly faster for P compared with TR, 2–slightly faster for P compared with TR, 3–identical for P and TR, 4–slightly faster for TR compared with P, and 5–markedly faster for TR compared with P. The overall software intuitiveness for both software types was rated by each observer applying a five-point Likert scale: 1 – markedly higher for P compared with TR, 2 – slightly higher for P compared with TR, 3 – identical for P and TR, 4 – slightly higher for TR compared with P, and markedly higher for TR compared with P. The issues impacting the workflow were described qualitatively. All procedures were performed with a commercial software (Prism 4.00, GraphPad Software, LaJolla, USA). Quantitative data were also expressed as mean and standard deviation with range. P<0.05 was considered as the level of statistical significance.

## Results

Liver segments II/III, II–IV and V–VIII served in 6, 3 and 7 donors as transplanted liver segments, respectively.

### Primary Study Goal

#### Intraoperative Weight and Volume of Transplanted Liver Segments

Full data are presented in Table 2. Volumes of transplanted liver segments were 642.9±368.8 ml for TR with vessels, 623.8±349.1 ml for P with vessels, and 605.2±345.8 ml for P without vessels. Statistically significant differences were detected between the 3 different techniques (P<0.01).

**Table 2:** Intraoperative Weights and Volumes of Transplanted Liver Segments.

Intraoperative weight (g)	TR volume with vessels (ml)	P volume with vessels (ml)	P volume without vessels (ml)	P-value*
636.1±363.1 (225.0–1310.0)*	642.9±368.8 (210.8–1345.0)*	623.8±349.1 (211.5–1281.0)*	605.2±345.8 (200.8–1291.0)*	<0.01

Note: *statistically significant differences between the 3 different techniques were evaluated applying ANOVA for repeated measures; mean of 4 reads (Read 1 and Read 2 for Observer 1 as well as Read 1 and Read 2 for Observer 2); given numbers are mean±SD (range).

#### Linear Regression Analysis between Intraoperative Weight and Volume of Transplanted Liver Segments

**Figure 2:**
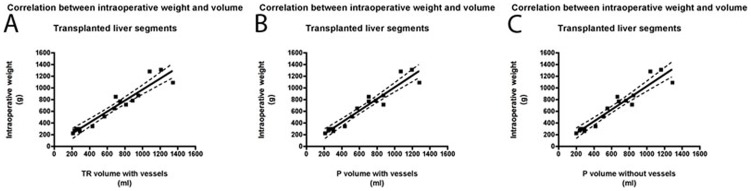
Linear Regression Analysis between Intraoperative Weights and Volumes of Transplanted Liver Segments. A For the manual commercial software (TR) with vessels, the regression equation was y = 0.94x+30.1 (R^2^ = 0.92; P<0.001). B For the semi-automated interactive software (P) with vessels, the regression equation was y = 1.00x+12.0 (R^2^ = 0.92; P<0.001). C For semi-automated interactive software (P) without vessels, the regression equation was y = 1.01x+28.0 (R^2^ = 0.92; P<0.001). Note: dotted curves mark the 95% confidence bands; linear regression analysis demonstrated a strong linear relationship between intraoperative weights and volumes with comparable results between the 3 different techniques.

Regression equations between intraoperative weights and volumes were y = 0.94x+30.1 (R^2^ = 0.92; P<0.001) for TR with vessels, y = 1.00x+12.0 (R^2^ = 0.92; P<0.001) for P with vessels, and y = 1.01x+28.0 (R^2^ = 0.92; P<0.001) for P without vessels (Fig. 2).

#### Disagreement between Intraoperative Weight and Volume of Transplanted Liver Segments

Error ratios were −1.5±14.2% (−21.7–20.2) for TR with vessels, −2.7±13.0% (−20.4–20.6) for P with vessels, and −6.5±14.1% (−27.3–17.2) for P without vessels. Statistically significant differences were detected between the 3 different techniques (P<0.01).

#### Intra-observer and Inter-observer Agreement of Volume of Transplanted Liver Segments

**Figure 3:**
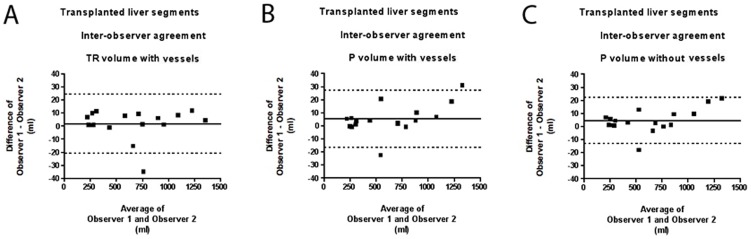
Blant-Altman Analysis for Inter-observer Agreement Regarding Volume of Transplanted Liver Segments. A Manual commercial software (TR) with vessels. B Semi-automated interactive software (P) with vessels. C Semi-automated interactive software (P) without vessels. Note: straight lines define bias; dotted lines define 95% limits of agreement; the inter-observer agreement can be regarded as “good” for the 3 different techniques.

Full data are presented in Table 3. Intra-observer agreement regarding volume for Observer 1/2 showed a bias of 25.9/−17.5 ml for TR with vessels, 2.4/−37.4 ml for P with vessels, and 5.1/−26.5 ml for P without vessels (Fig. 3). Inter-observer agreement regarding volume showed a bias of 1.8 ml for TR with vessels, 5.4 ml for P with vessels, and 4.6 ml for P without vessels. For inter-observer agreement regarding volume, the 95% limits of agreements were −20.6–24.4 ml for TR with vessels, −16.6–27.4 ml for P with vessels, and −12.8–22.1 ml for P without vessels.

**Table 3:** Intra-observer and Inter-observer Agreement of Volume of Transplanted Liver Segments.

TR volume with vessels (ml)			P volume with vessels (ml)			P volume without vessels (ml)		
Intra-observer agreement		Inter-observer agreement	Intra-observer agreement		Inter-observer agreement	Intra-observer agreement		Inter-observer agreement
O 1	O 2	O1* versus O2*	O 1	O 2	O1* versus O2*	O 1	O 2	O1* versus O2*
25.9 (−37.8–89.5)	−17.5 (−72.0–37.0)	1.8 (−20.6–24.4)	2.4 (−99.9–104.8)	−37.4 (−163.3–88.6)	5.4 (−16.6–27.4)	5.1 (−85.4–95.7)	−26.5 (−115.5–62.4)	4.6 (−12.8–22.1)

Note: O for Observer; intra-observer and inter-observer agreement was evaluated applying the Blant-Altman analysis: bias (95% limits of agreement); *mean of Read 1 and Read 2 of Observer 1 and Observer 2, respectively; given numbers are mean±SD (range).

### Secondary Study Goal

**Figure 4:**
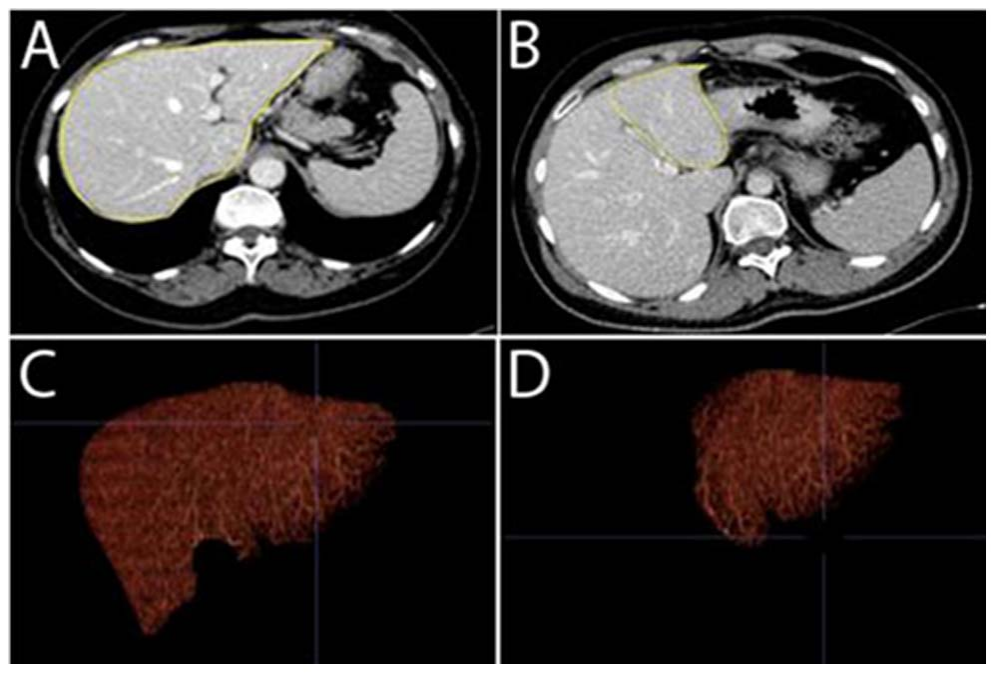
Manual Commercial Software (TR) – Image Example. A Transverse image of the portal-venous phase – manual outline of the entire liver (yellow). B Transverse image of the portal-venous phase – manual outline of liver segments II/III (yellow). C Volume rendering (coronal view) resulting after manual outline of the entire liver. D Volume rendering (coronal view) resulting after manual outline of liver segments II/III. Note: in each live liver donor, CT-volumetry of the entire liver as well as of the future liver graft (transplanted liver segments) were performed to ensure that the postoperative liver volume is adequate.

**Figure 5:**
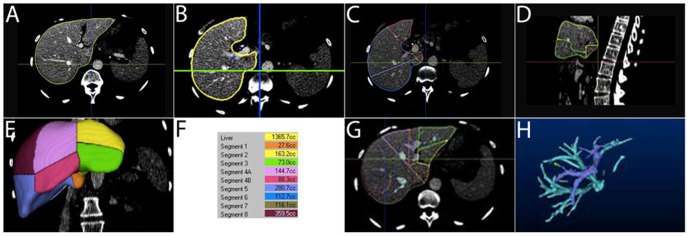
Semi-automated Interactive Software (P) – Image Example. A Transverse image of the portal-venous phase – automated outline of the entire liver after manual correction of false-positive and false-negative extractions. B Manual positioning of the anatomical landmark “first bifurcation of the right portal vein” (blue circle) according to Fig. 1A. C Automated definition of segments of Couinaud for right liver - transverse image. D Automated definition of segments of Couinaud for left liver - sagittal image. E Volume rendering (coronal view) with automated definition of segments of Couinaud of the entire liver. F List of volumes for the different segments of Couinaud. G Transverse image of the portal-venous phase – automated outline of the entire liver after manual correction of false-positive and false-negative extractions. H Volume rendering (coronal view) with automated definition of vessels (liver veins in light blue and portal veins in dark blue). Note: in each live liver donor, CT-volumetry of the entire liver was performed to ensure that the postoperative liver volume, calculated on the basis of Fig. 5F, is adequate.

For the degree of manual interaction, the mean scale value was 2.6±0.8 (1–5) (Fig. 4, 5). Accordingly, the degree of manual interaction was slightly lower for P compared with TR. For TR, the process of manual outline of the liver applying the free ROI tool was perceived as the major reason for the higher degree of interaction for TR. For P, manual correction tools were used on average in 8 reads (6–10 reads) per series (each series consisting of 16 reads) for correction of too large or too small Couinaud segments. For the time for completion of CT-volumetry, a scale value of 2.4±0.5 (1–5) resulted. Accordingly, the speed for completion of CT-volumetry was faster for P compared with TR. For TR, uploading of images and manual outline of the liver applying the free ROI tool were perceived as the most time consuming steps. Subsequent calculation of volume of transplanted liver segments was very fast. For P, uploading of images was perceived as a time consuming step, whereas automatic outline of the entire liver was fast. The time required for the correction of false-positive and false-negative extractions was on average 3 min (1–8) for the reads with the use of manual correction tools. The latter was also perceived as the overall most time consuming step for P. The time necessary to position the landmarks in the “work-me-through” tool was on average 2 min (1-4). Subsequent calculation of the volume of transplanted liver segments was very fast, irrespective of whether the vessels were considered or not. For the overall software intuitiveness, both observers rated a scale value of 2. Accordingly, the software intuitiveness was rated slightly lower for TR compared with P. For TR, software intuitiveness was perceived “good”, and both observers reported multiple years experience with this software. For P, the “work-me-through” tool was perceived “very helpful”, and the clinical implementation of this new software was perceived “auspicious”.

## Discussion

In this study, accuracy of estimation of graft size for living-related liver transplantation was evaluated using a semi-automated interactive software (P), and compared with a manual commercial software (TR). Prediction of graft size was good with strong linear relationships and low error ratios between intraoperative weights and volumes for the different transplanted liver segments. Inter-observer and intra-observer agreements were good. Compared with TR, the workflow was better for P. The results confirmed our hypothesis that CT-volumetry performed with P can accurately predict graft size while improving workflow compared with TR.

Yoneyama et al. estimated the liver graft weight from preoperative CT [17]. The coefficient factor between estimated graft volume and actual graft weight was 0.84 for right lobes and 0.85 for left lobes. Their data indicate that CT-volumetry overestimated the actual graft weight. In our study, there was a slight trend of underestimation of the intraoperative weight of transplanted liver segments. In this context, the different approaches for CT-volumetry should be mentioned. In the study of Yoneyama et al., the volume was calculated automatically by summation of the products of section thickness and area in each section of the segmented liver. In our study, CT-volumetry was performed as voxel analysis within the outlined liver. Li et al. published results for CT-volumetry in left lobe liver donation [3]. The outline of the future liver graft was traced and marked section by section by means of a cursor with manual exclusion of non-parenchymal structures (e.g. portal vein). They found an error ratio of 13.8±8.1%, and as in the study by Yoneyama et al., the graft weight was overestimated although vascular structures were not segmented. From our 3 different techniques, P without vessels showed the largest differences between intraoperative weight and volume. It is likely that the significant differences of Table 2 result from this observation (since the results for TR and P with vessels were comparable). We recommend therefore to use P with vessels for the preoperative evaluation of the graft volume for potential liver donors.

Kim et al. performed a study with 88 living-related liver donors applying automated blood-free CT-volumetry [2]. The five main steps of this automated software consisted of “pre-processing”, “initial shape detection”, “liver segmentation”, “vessel segmentation” and “liver resection”. The authors found a CT volume of 789.0±126.4 ml for blood-filled right lobe and 713.9±114.4 ml for blood-free right lobe, whereas the intraoperative weight was 717.8±110.4 g. The slight underestimation of the graft weight according to blood-free CT-volumetry is in line with our results. Kim et al., however, found the best prediction of graft weight for blood-free CT-volumetry. The corresponding linear regression equation was y = 0.88x+88.5 (R2 = 0.83; P<0.001). Our results for linear regression analysis demonstrated also a strong linear relationship between intraoperative weights and volumes, with comparable results between the 3 different techniques.

For intra-observer and inter-observer agreement regarding CT-volumetry, there is a lack of data in living-related liver donation. Our results for inter-observer agreement regarding volume were excellent, with a maximum bias of 5.4 ml. For intra-observer agreement of volume, a maximum bias of −37.5 ml was found. In view of the absolute values, this agreement could be rated as “clinically acceptable”. The latter is also encouraged by the study of Dello et al., who discussed that mean liver resection volume differences of 62.3 ml (987.7±64.0 ml for Surgeon 1 and 1050.0±78.6 ml for Surgeon 2 applying the same software) should have no clinical consequences [18]. This statement is particularly remarkable as the weight of the resected specimen (788.8±53.7g) was lower by approximately one fourth.

The different software algorithms available for CT-volumetry could also impact significantly the accuracy of estimation of future graft size. A word of caution, however, should be given to the reference standard “intraoperative weight”. According to the publication of Satou et al., back-table procedures can affect the weight of transplanted liver segments [9]. During surgical preparation, the graft weight decreased significantly from “blood-filled” over “blood-free” over “after perfusion” to “after venoplasty”. The authors discussed dehydration effects (e.g. induced by high osmotic preservative solution) and preparation (e.g. trimming for venous reconstruction) as relevant. Another interesting point to discuss is the perfusion pressure. Müller et al. analyzed liver volumes in an experimental setting [19]. Ten pig livers were studied in-vivo, and additionally in an ex-vivo perfusion simulator. The deviation for perfused and non-perfused livers applying ex-vivo CT-volumetry was 22.9% (15.5–37.8). The paired in-vivo results applying the water displacement technique were comparable, with a deviation for perfused and non-perfused liver volumes of 22.9% (19.0–25.6). Those observations should be kept in mind when in-vivo (perfused) CT-volumetry is compared with ex-vivo (non-perfused) gravimetry.

The workflow with a special focus on CT slice thickness was analyzed by Hori et al. [20]. The mean time required for completion of the volumetric analyses was 98 min. Although four segmentations were included (four different slice thicknessess), the time necessary per segmentation seems to be markedly longer compared with our study. Dello et al. concluded that a slice thickness of 10 mm provides an optimal balance between accuracy and time efficiency [18]. On the contrary, Puesken et al. published that a CT slice thickness of no more than 3mm should be used because of the significant deviations in measurements for thicker slices [21]. The dependency between user interaction and the different software algorithm was published by Zhou et al. [22]. As one result, the more tasks are shifted to the software, the more degrees of freedom are introduced and the larger the variations that occur. In our study, the degree of manual correction/interaction was lower for P compared with TR. Both observers reported some degree of freedom for the positioning of the anatomical landmarks (e.g. multiple slices fulfilled the landmark criteria of Fig. 1). It can be speculated that further specification in the “work-me-through” tool could lead to better volumetric results. Finally, the learning curve was better for P which is not surprising since both observers were accustomed to use TR for years. While for TR, the procedural steps regarding time and accuracy were ”almost identical” between Read 1 and Read 2, for P both observers were more efficient in Read 2.

This study has limitations, most important is the small number of living-related liver donors. Although the potentially low statistical power, our concept showed feasibility in this first-in-man analysis, and opened the way to analyze more clinical data. Secondly, the absolute time to perform CT-volumetry was not recorded systematically, and therefore the description of representative quantitative data is impossible. In order to still indicate results for the duration of CT-volumetry, we used semi-quantitative methods in the form of a Lickert scale.

In conclusion, CT-volumetry performed with P can accurately predict graft size for living-related liver transplantation while improving the workflow compared with TR. The clinical use of the presented semi-automated interactive software might facilitate the radiological routine without reducing reporting quality.
